# Powerful tool or too powerful? Early public discourse about ChatGPT across 4 million tweets

**DOI:** 10.1371/journal.pone.0296882

**Published:** 2024-03-27

**Authors:** Reuben Ng, Ting Yu Joanne Chow

**Affiliations:** 1 Lee Kuan Yew School of Public Policy, National University of Singapore, Singapore, Singapore; 2 Lloyd’s Register Institute for the Public Understanding of Risk, National University of Singapore, Singapore, Singapore; Institut Teknologi Bandung, INDONESIA

## Abstract

**Background:**

This paper investigates initial exuberance and emotions surrounding ChatGPT’s first three months of launch (1 December 2022–1 March 2023). The impetus for studying active discussions surrounding its implications, fears, and opinions is motivated by its nascent popularity and potential to disrupt existing professions; compounded by its significance as a crucial inflexion point in history. Capturing the public zeitgeist on new innovations—much like the advent of the printing press, radio, newspapers, or the internet—provides a retrospective overview of public sentiments, common themes, and issues.

**Objectives:**

Since launch, few big data studies delved into initial public discourse surrounding the chatbot. This report firstly identifies highest-engagement issues and themes that generated the most interaction; secondly, identifies the highest-engaged keywords on both sides of the sentiment valence scale (positive and negative) associated with ChatGPT.

**Methods:**

We interrogate a large twitter corpus (n = 4,251,662) of all publicly available English-language tweets containing the ChatGPT keyword. Our first research aim utilizes a prominent peaks model (upper-quartile significance threshold of prominence>20,000). Our second research aim utilized sentiment analysis to identify, week-on-week, highest-frequency negative, and positive keywords and emojis.

**Results:**

Six prominent peaks were identified with the following themes: ‘hype and hesitance’, ‘utility and misuse in professional and academic settings’, ‘demographic bias’, ‘philosophical thought experiments on morality’ and ‘artificial intelligence as a mirror of human knowledge’. Of high-frequency valence, negativity included credibility concerns, implicit bias, environmental ethics, employment rights of data annotators and programmers, the ethicality of neural network datasets. Positivity included excitement over application, especially in coding, as a creative tool, education, and personal productivity.

**Conclusions:**

Overall, sentiments and themes were double-edged, expressing excitement over this powerful new tool and wariness toward its potential for misuse.

## Introduction

Since the introduction of ChatGPT—a natural language processing chatbot developed by the company OpenAI—on December 1, 2022, its userbase exploded in popularity, netting a hundred million in its first month. While being the most popular, ChatGPT is only one out of many other generative programs that are powered by processing models: among generative art programs like *DALL-E* and *Stable Diffusion*; representing a crest in a wave of new artificial intelligence technology arriving on the shores of the internet. As the world stands on the precipice of new innovations—the advent of the printing press, the radio, newspapers, for instance—they often catch their share of ire and praise. In similar fashion, ChatGPT has generated a flurry of discussions on its implications and potential uses.

The burgeoning impact of ChatGPT has been investigated from an academic lens, ranging from disapproval over its dubious role in research authorship [1], difficulties in discerning human-generated abstracts from ChatGPT-generated abstracts [2]; though the technology may democratize the playing field by aiding weaker writers [3], with one in three healthcare researchers skewing positive on its applications [4]. Its ramification on medical scholarship is pronounced, particularly in instances where it confidently creates false facts [5], or creates outputs rife with inaccuracies [6]; suggesting that a degree of caution and human judgment must be exercised [7] despite its strengths [8]. While many such studies, editorials, and commentaries have been published in relation to ChatGPT’s impact in professional settings, fewer exist in relation to general perceptions. Since its launch, few big data studies have delved into initial public discourse surrounding the chatbot. Existing large-scale data studies on early adopters suggest overwhelmingly positive sentiment toward the technology [9], though fears surround its impact on existing jobs [10]; early sentiments involve excitement on its potential applications, albeit with red flags on ethical concerns [11]. Other studies have also found that ChatGPT, when used as a chatbot, generated higher-quality and more empathetic replies than the average professional [12], and was met with enthusiasm over its applications in education [13] and its user-friendly interface as an information amalgamator [14]. Such technologies have the potential to cut a swathe through existing professional sectors, thus presenting a crucial inflexion point in history to capture the public zeitgeist on artificial intelligence.

With this impetus established, we aim to analyze the exuberance and emotions surrounding ChatGPT’s initial public perceptions. We interrogate a corpus dataset of all English-language tweets containing the *ChatGPT* keyword from 1 December 2022 to 1 March 2023 (n = 4,251,662) to ask two research questions: First, *what are the biggest issues or themes that generated the most engagement*? Second, *what are the highest-frequency keywords*, *and what are its sentiments like*? Our first aim is achieved by running a prominent peaks model (prominence > 20,000), identifying themes associated with mention count spikes. Our second aim identified highest-frequency keywords rated by valence (positive, neutral, negative), mapped chronologically by week. By focusing on metrics that generated the most discussion, we provide a bird’s eye view of burgeoning narratives that surrounded this new technology’s debut onto the global stage.

## Methods

### Dataset

**Fig 1:**
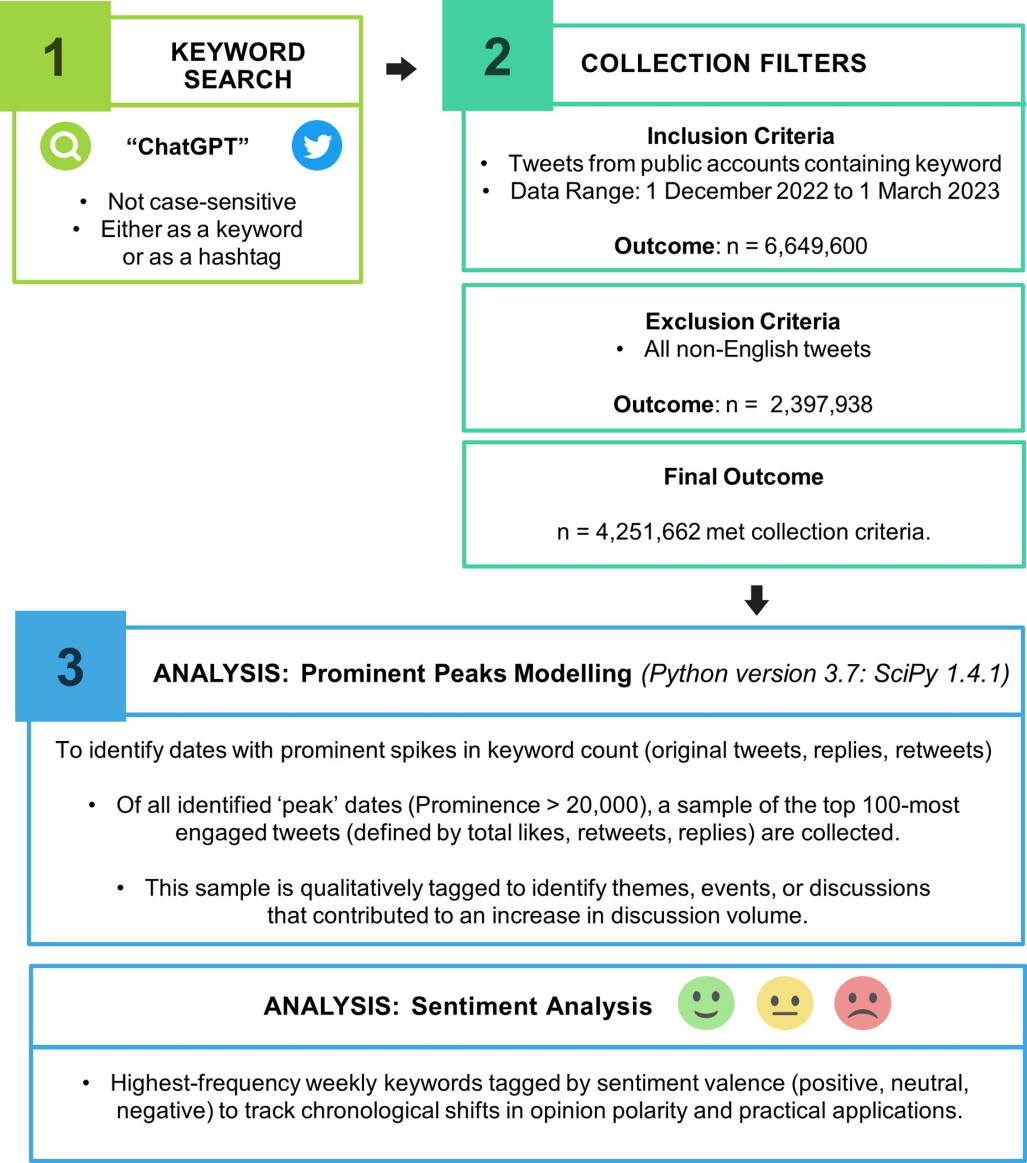
Methodological summary: Data collection and analytic plan.

The dataset used for this study was collected using Twitter’s application programming interface standard search, publicly accessible via Twitter API V2 on the academic research access level. All English-language tweets containing the ‘ChatGPT’ keyword—used in context as either a keyword or hashtag, and not case-sensitive—published between 1 December 2022 and 1 March 2023 were shortlisted (n = 4,251,662). A summary of methods used is available in Fig 1.

### Research aim 1: Prominent peaks modelling

Our first research aim involved two methodological steps. First, we quantitatively identified prominent peak dates in which ‘ChatGPT’ experienced a spike in mention count—defined by the total number of original tweets, replies, and retweets containing the keyword—signaling a significant increment in interest and engagement in the topic. This analysis was conducted using peak prominence detection modelling (Python version 3.7: SciPy 1.4.1).The python package module operated based on marking the higher of two bases as the peak’s lowest contour line. Prominence was then calculated as the vertical difference between the peak’s height, and its lowest contour line. Simply put, for any given date with a certain mention value (A), the program checks for the lowest possible point *prior* to that date (B), and likewise in the other chronological direction for the lowest possible point *after* that date (C): the average of these two values (A-B; A-C) is then defined as the date’s prominence score, a value assigned relative to the lowest possible point on either side of the calendar.

A total of 23 statistically significant peaks were found during the study period (x̄ = 17,564; σ = 21,883); of which, prominence scores of said peaks were calculated to range from 4582 (lower quartile), to 8932 (median), to 20838 (upper quartile). Using a data-driven approach to determine an adequate threshold for significance, we selected peaks that appeared only in the upper quartile of prominence (i.e., Prominence > 20,000) for further investigation, essentially, delving into the top 75% of discussion spike dates. This method yielded 6 peaks: 6 individual dates where prominence exceeded the upper quartile’s significance threshold.

**Fig 2:**
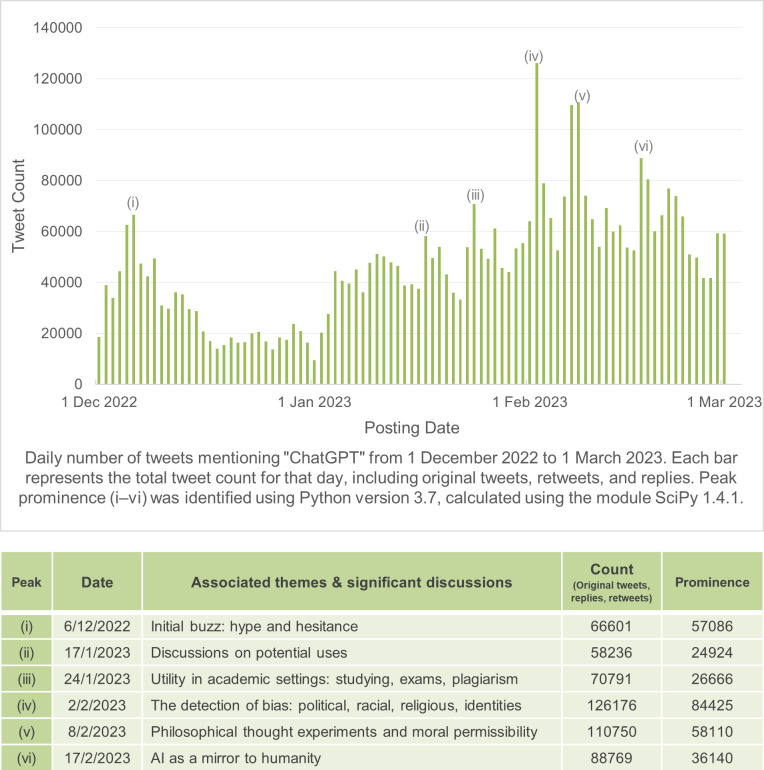
Prominent peaks in ChatGPT conversations across 4 million tweets.

Using these identified 6 upper quartile peak dates, our second methodological step involved qualitatively sampling the top engaged tweets on those days (i.e., a qualitative interrogation of the retweets, replies, and discussions, and a sample of the top-100 tweets with the most ‘likes’), to identify the main themes, events, or discussions that contributed to the day’s significant increase in discursive volume, taking into account the detractors (often represented by quote tweets or replies) and supporters (often represented by retweets and large amounts of ‘likes’) of certain topics or emergent issues. A summary of results is presented in Fig 2.

### Research aim 2: Weekly top keywords and valence

Our second research aim chronicled highest-frequency keywords used in conjunction with ChatGPT tweets, further subcategorized by valence (positive or negative sentiment polarity) or practical utility (neutral sentiment, keywords about potential uses) on a weekly basis. This involved two methodological steps. The first step involved identifying frequently co-occurring collocates (individual words, bigrams, trigrams, quadrigrams: scored by raw frequency totaling retweets, reposts and unique mentions) in our corpus dataset. Second, these high-frequency keywords were then evaluated for their sentiment valence (positive or negative) and whether the phrase related to potential uses (neutral) and sorted into **Table 1**, along with the top five emojis used in that week. This analysis provides a rolling chronology of shifts in opinions: term frequency was used as a proxy for overall public buzz, representing the main sources of interactions and discussions about practical applications and the ethics of AI during this early adoption window.

**Table 1:** Highest frequency keywords by sentiment valence.

Week (Date Range)	Sentiment Valence and Respective Keywords			
	Negative	Positive	Neutral	Top 5 Emojis (Unicode v15.0 CLDR Short Name)
**1** (30/11/22–04/12/22)	• crazy chatgpt loop • hallucinated chat room • weird trick • exactly zero coverage	• huge breakthrough • good debugging companion • biggest tech innovation	• proprietary prompt • new ai system • bubble sort algorithm • technical questions • senior data engineer	• exploding head • repeat button • thread • face with tears of joy • backhand index pointing down
**2** (05/12/22–11/12/22)	• problem of bias • misleading impression • blockchain trilemma • cognitive dissonance	• robustness and truthfulness • unique human skill • full potential	• workout plan • meal plan • calorie targets • unique architecture • modern physics	• thread • eyes • backhand index pointing down • laptop • exploding head
**3** (12/12/22–18/12/22)	• stolen artwork • absolute minimal effort • nonsense • overconfidence • get rich-quick scheme	• best possible use	• large language models • illustrator • al image generator	• thread • backhand index pointing down—face with tears of joy • fire • exploding head
**4** (19/12/22–25/12/22)	• fossil fuels • variants and competitors	• fascinating applications	• lesson plans • code • recent investments • nuclear energy	• thread • backhand index pointing down • exploding head • backhand index pointing right • face with tears of joy
**5** (26/12/22–01/01/23)	• counter-suggestions • ai explosion	• possibilities • internet by storm • new era	• copywriters • open-source implementation • whole new industry • many creators	• rocket • robot • backhand index pointing down • winking face • thread
**6** (02/01/23–08/01/23)	• limited knowledge • simple mistakes	• insanely useful • powerful tools • incredible ways	• modern marketing • daily workflow	• backhand index pointing down • exploding head • thread • link • police car light
**7** (09/01/23–15/01/23)	• religious bias	• leverage • full potential • productivity • best open-source speech model • free marketing assistant • eye-openers	• creators and writers • speech • essays • velocity and quality • value of inclusion • best open-source speech model	• backhand index pointing down • thread • exploding head • fire • grinning face with smiling eyes
**8** (16/01/23–22/01/23)	• outsourced kenyan workers • shocking twist • investigation	• free employee • magical computer intelligence	• education • marketing assistant	• police car light • thread • backhand index pointing down • fire • robot
**9** (23/01/23–29/01/23)	• magic nothingness	• amazing resource • free assistant	• charm and syntax • ambiguous scenarios • undergraduate paper • med school	• thread • backhand index pointing down • exploding head • face with tears of joy • robot
**10** (30/01/23–05/02/23)	• wing bias • credibility damage • irreparable	• content creation superpower	• grammar checker • homework • academic purposes • poem • natural language processing engine • personalized meeting templates	• backhand index pointing down • collision • exploding head • blue book • police car light
**11** (06/02/23–12/02/23)	• political bias • racial slur • pithy essay	• phenomenal ai • super tool • full potential	• best chatgpt resources • chatgpt for advice • scientific paper • sharing of training data • students	• backhand index pointing down • thread • rocket • fire • backhand index pointing right
**12** (13/02/23–19/02/23)	• unbelievably woke response • gaslights	• ai memes • active community	• finance research • freelancer and businessman • job advert	• fire • check mark button • backhand index pointing down • robot • backhand index pointing right
**13** (20/02/23–26/02/23)	• fake citations to papers • 5th-gen warfare tools • flaws	• trust and safety • engaging content	• basic marketing • academic writing • copywriting tools	• robot • backhand index pointing down • fire • check mark button • partying face

## Results

### Overall peaks and emergent issues

We present findings of identifying prominent peaks and emergent issues within the first three months of ChatGPT’s official release in Fig 2. Peak prominence was observed in dates (i)–(vi), with each spike attributable to a new dominant discoursal theme.

#### Peak (i): Initial buzz: Hype and hesitance

This first peak was attributable to the technology’s nascent popularity: this day was met with excitement about its userbase crossing a million users 5 days into launch. Positive sentiments included speculation on its value to creative fields as a content-generation aid; merits as an amalgamator of existing human knowledge; its potential to usurp existing tech goliaths; and the relative ease and accessibility for users to submit prompts. This was countered by skepticism: the bot criticized for its tendency to confidently present nonfactual data as fact (e.g., non-existent citations, untrue scientific information, providing different answers to similar prompts); its credibility was problematized in phrases like ‘*hallucinations’*, ‘*echo chamber’* and ‘*black box’* to describe inconsistent data outputs.

#### Peak (ii): Discussions on potential uses

This peak surrounded discourse on how the technology was viable for increasing efficiency in professional fields like business or consumer marketing, enhancing personal productivity, and in simplifying basic tasks. Within this peak, several tech accounts and bots were observed to promote links to full guides, crash courses, and tutorial resources on search prompt optimization.

#### Peak (iii): Utility in academic settings: Studying, exams, plagiarism

This peak was related to academia. Discussions surrounded whether academic essays produced by the bot were discernible from the average student: one camp of educators reflected it was almost impossible to tell, while another claimed that paragraphs produced by the bot—while grammatically accurate—lacked the earnestness and complex syntax of authentic student papers. Mild panic ensued over the bot’s ability to obtain passing grades on examinations in knowledge-based fields (e.g., law, medicine, business); though this was countered by clarifications that such tests were often multiple-choice—not written—and entirely divorced from actual professional practice. The issue of plagiarism was also discussed: some likened the bot’s assistive function as a research and proofreading tool to the use of calculators in modern-day math classes; while others believed its use lacked academic integrity.

#### Peak (iv): The detection of bias: Political, racial, religious, identity markers

This peak involved criticism on the inconsistent output of controversial prompts. Demographic bias surfaced along the lines of political, racial, religious, gender- and sexual-identities, with the bot refraining from generating content about certain groups over others. Debates ensued over the degree to which AI engineers held responsibility over this issue of unequal treatment. Users opined on whether this was deliberately programmed malicious propaganda, or simply shortcomings of any new technology to be improved upon with time; suggesting that artificial systems should behave descriptively rather than prescriptively: neutral by default, but with parameters allowing individual user customization and preference toggles.

#### Peak (v): Philosophical thought experiments and moral permissibility

This peak was dominated by a trend of submitting thought experiments as prompts: for instance, presenting the ‘trolley problem’ or ‘train car experiment’—common philosophical conundrums presenting an ethical dilemma. Common iterations included presenting the bot with hypothetical choices: between killing one person or five; saving young children or older adults; using a racial slur or setting off an atomic bomb. Others also elicited nuanced ethical judgments from the bot by asking complex philosophical questions on human nature and war. Others attempted to circumvent off-limits questions by setting specific prompt parameters, thereby granting the bot ‘permission’ to take on different personas by answering questions as separate hypothetical entity unbeholden to coded restrictions on sensitive topics. Submitters of these prompts would then point to the bot’s generated answer as a sign of implicit bias. Conversely, other users pointed out that the bot functioned by mimicking patterns in human language using the dataset it was trained on, and it was thus futile to ascribe morality to its outputs.

#### Peak (vi): AI as a mirror to humanity

This engagement peak saw a flurry of discussions about the nature of artificial intelligence holding a mirror up to humanity, representing the massive collection of texts its neural network is trained on. Parallels were drawn between the bot as a more sophisticated ‘auto-complete’ function, due to its output reflecting the most statistically probable series of words based on a collective set of human knowledge. Others described this technology akin to a less-impressive parrot: only repeating data it was trained on and constricted further by alleged censorship coded by AI engineers who set restrictions on topics deemed too sensitive.

### Top keywords by valence

We present findings of top keywords rated by sentiment score in Table 1, mapping weekly chronological progressions of highest-frequency terms.

Overall, highest-frequency negative keywords indicate concerns about credibility of the new technology (e.g. *hallucinated*, *crazy loop*, *cognitive dissonance*, *limited knowledge*, *simple mistakes*, *overconfidence*, *misleading*), implicit bias when generating answers for queries (e.g. *bias*, *misleading*, *political bias*, *wing bias*, *religious bias*), environmental ethics (e.g. *fossil fuels*), employment rights of data annotators (e.g. *outsourced workers*, *investigation*), and adjacent debates on whether programs based on a neural network of existing human works is ethical (e.g. *stolen artwork*, *minimal effort*).

Highest-frequency positive and neutral keywords indicate excitement over general possibilities (e.g., *huge breakthrough*, *biggest tech innovation*), especially in coding (e.g., *good debugging companion*, *insanely useful*, *code*) and as a creative tool (e.g., *content creation superpower*, *copywriters*), education (e.g., *lesson plans*, *essays*, *undergraduate paper*, *academic purposes*, *grammar checker*), daily personal use (e.g., *workout plan*, *meal plan*, *calorie targets*, *personalized meeting templates*)

The highest-frequency emojis generally point to excitement and interest over the new technology (e.g., ‘mind-blown/exploding head’ [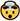], ‘attentive eyes’ [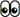], and conveying something being explosively exciting (e.g., ‘boom/collision’ [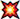], ‘fire’ [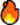]), technological applications (e.g., related to technology and information: laptop, rocket, robot, blue notebook [
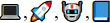
]), sharing of information in threads (e.g., ‘call-to-action’ emojis prompting users to click for more: thread, backhand index fingers pointing downward or to the right, embedded hyperlinks [
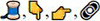
]), caution and ridicule (e.g., generally conveying doubt: ‘laughing-crying face with tears of joy’ [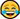], ‘alarm bell/police car light’ [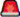]).

## Discussion and conclusion

We analyzed large-scale data of emergent public chatter, focusing on engagement and frequency metrics surrounding ChatGPT’s initial launch. Our research findings indicate discussions around its myriad uses, particularly in education and professional settings, though tempered by doubt over its accuracy. This burgeoning technology raised questions on the ethics of artificial intelligence, surfacing issues of implicit demographic bias and philosophical dilemmas on morality judgements. Summarily, prominent peak analysis suggests excitement surrounding the potentially powerful tool, met with doubt due to a potential for misuse—perfectly emblematizing initial hype and hesitance.

Future research may involve tracking the chronological progression of this technology, charting public perception as the software is improved. It is also acknowledged that ChatGPT is not the only publicly available AI model: follow-up analysis using the methodology may be applied to track public perceptions on other popular generative models (e.g., *DALL-E*, *Stable Diffusion*).

Of conceptual significance, this paper contributes to building a structured understanding of social media narratives of an explosive new technology: an artificial intelligence tool that entered public consciousness with a splash. A record of the early narratives of such a historic event provides a referential basis upon which future new phenomena could be compared. By that token, of methodological significance is the replicability of the methodology across different contexts, sectors, or industries. The methodology may also be replicated for the same ChatGPT topic across a future time-period, to compare whether societal narratives on such technologies have become more nuanced, or changed dramatically alongside new events. Of practical significance, such studies deepen our understanding of how people react to new technologies—worries and woes on ethical concerns—which may inform how tech companies could consider framing new releases to prevent unintentional backlash.
